# High expression of TRF2, SOX10, and CD10 in circulating tumor microemboli detected in metastatic melanoma patients. A potential impact for the assessment of disease aggressiveness

**DOI:** 10.1002/cam4.661

**Published:** 2016-03-06

**Authors:** Elodie Long, Marius Ilie, Coraline Bence, Catherine Butori, Eric Selva, Salomé Lalvée, Christelle Bonnetaud, Gilles Poissonnet, Jean‐Philippe Lacour, Philippe Bahadoran, Patrick Brest, Eric Gilson, Robert Ballotti, Véronique Hofman, Paul Hofman

**Affiliations:** ^1^ Institute for Research on Cancer and Aging in Nice (IRCAN) INSERM U1081/CNRS UMR7284University of Nice Sophia AntipolisAntoine Lacassagne Cancer CenterNiceFrance; ^2^ Laboratory of Clinical and Experimental PathologyPasteur HospitalNiceFrance; ^3^ Human BiobankPasteur HospitalBB‐0033‐00025NiceFrance; ^4^ Department of SurgeryComprehensive Cancer CenterAntoine LacassagneNiceFrance; ^5^ Department of DermatologyArchet II HospitalNiceFrance; ^6^ INSERM U1065 Team 1University of Nice Sophia AntipolisEquipe Labellisée Ligue 2013NiceFrance; ^7^ Unit of GeneticsArchet HospitalNiceFrance; ^8^ Cancer Research Association (ARC) Labelled TeamVillejuifFrance; ^9^ “OncoAge” Hospital‐University FederationCHU NiceFrance

**Keywords:** Circulating tumor cells, circulating tumor microemboli, immunocytochemistry, Metastatic melanoma, prognosis

## Abstract

Circulating tumors cells (CTCs) can be detected in the blood of metastatic melanoma patients (MMPs) both as isolated circulating tumor cells (iCTCs) and circulating tumor microemboli (CTMs), but their clinical significance remains unknown. The aim of this work was to evaluate the prognostic impact in metastatic cutaneous melanoma of CTMs and iCTCs identified by a cytomorphological approach using the isolation by size of tumor cell (ISET) method. We characterized the phenotype of CTCs using anti‐PS100, anti‐SOX10, anti‐CD10, and anti‐TRF2 antibodies. 128 MMPs and 37 control healthy individuals with benign nevi were included in this study. Results were compared to the follow‐up of patients. 109/128 (85%) MMPs showed CTCs, 44/128 (34%) with 2 to 6 CTMs and 65/128 (51%) with 4 to 9 iCTCs. PS100 expression was homogeneous in iCTCs and heterogeneous in CTMs. SOX10, CD10, and TRF2 were mainly expressed in CTMs. None of the control subjects demonstrated circulating malignant tumor cells. Overall survival was significantly decreased in patients with CTMs, independently of the therapeutic strategies. In conclusion, the presence of CTMs is an independent predictor of shorter survival from the time of diagnosis of MMPs.

## Introduction

The fluid biopsy concept in the oncology field is opening new and exciting perspectives for optimization of the care of cancer patients in using a noninvasive approach 1, 2. In this context, the detection of circulating tumor cells (CTCs) in metastatic melanoma patients (MMPs) should allow, (1) better monitoring of patients, (2) the detection of genomic alterations, which can be accessible to targeted therapies, (3) the identification of secondary‐resistant mutations, and (4) the establishment of new prognostic factors 3, 4, 5. Moreover, the molecular characterization of CTCs should increase our knowledge into the pathophysiology and the natural history of malignant melanoma, in particular in metastatic dissemination. Finally, working in the fluid biopsy field will lead investigators to develop new therapeutic strategies for MMPs 6, 7.

Many direct and indirect methods can be used for detection of CTCs in MMPs 8, 9, 10, 11. Among them, the isolation by size of epithelial tumor cells technology (ISET) allows both morphological identification and phenotypical characterization of circulating tumor (melanoma) cells 12, 13, 14, 15, 16.

The numbers of CTCs has been shown to be prognostic of overall survival in MMPs 14, 16. Moreover, using different biomarkers, such as the melanoma‐associated markers MCSP (melanoma‐associated chondroitin sulfate proteoglycan) and MCAM (melanoma cell adhesion molecule) and the melanoma initiating cell markers ABCB5, CD271, and RANK, Gray and collaborators have identified CTC subpopulations in MMPs 17. Moreover, they demonstrated the potential prognostic value of these CTC subpopulations 17.

The CTCs can be detected in carcinoma and melanoma patients as isolated CTCs (iCTCs) and/or as aggregates of variable numbers of circulating tumor cells, namely circulating tumor microemboli (CTMs). The phenotype of tumor cells of CTMs can be heterogeneous and can differ from that observed for iCTCs, which may reflect potential higher cell aggressiveness and invasiveness. However, the clinical significance and the molecular characterization of CTMs in MMPs have been poorly and partially investigated to date.

The purpose of this work was to quantify CTMs in MMPs using the ISET method and to evaluate the respective clinical significance of iCTCs and CTMs. Moreover, according to previous studies that demonstrated the potential role of different molecules (PS100, SOX10, TRF2, CD10) in the aggressiveness and differentiation of cutaneous melanoma, we characterized the phenotype of the different circulating melanoma cell subpopulations using anti‐PS100, anti‐SOX10, anti‐TRF2, and anti‐CD10 antibodies 18, 19, 20, 21, 22, 23.

## Materials and Methods

### Patients

One hundred and twenty‐eight consecutive patients with metastatic melanoma were entered into this study at the University of Nice Sophia Antipolis (Nice, France) from September 2007 to January 2014. Follow‐up of these patients was from 3 to 18 months (mean 12 months). 63/128 (49%) patients received targeted anti‐BRAF inhibitor therapy (vemurafenib) and 65/128 (51%) patients received dacarbazine therapy. A blood sample was taken before the first‐line treatment. Additionally, 21 patients with benign nevi and 16 control healthy individuals were included in this study. All patients gave their informed consent to participate in this study. The study was conducted according to Good Clinical Practice and the Declaration of Helsinki. The main clinicopathological parameters are summarized in Table 1. Results correlated with the follow‐up of patients, and more specifically the overall survival (OS).

**Table 1:** Main clinico‐pathological parameters of the MMPs included in this study

Variables	Patients n (%)
Gender	
Male	78 (61%)
Female	50 (39%)
Age (years)	
Median (min – max)	57 (18 – 85)
Primary tumor site	
Head and neck	24 (19%)
Limbs	54 (42%)
Trunk	29 (22%)
Hands or feet	15 (11%)
Unknown	8 (6%)
Histology	
SSM	34 (27%)
NM	65 (51%)
LMM	5 (4%)
ALM	12 (9%)
Others	12 (9%)
Ulceration	
Absent	30 (23%)
Present	98 (77%)
Metastatic sites	
Regional lymph nodes	32 (25%)
Lung	13 (10%)
Brain	12 (9%)
Liver	6 (5%)
Disseminated	65 (51%)
AJCC staging	
IIIb	17 (13%)
IIIc	23 (18%)
IV	88 (69%)
M1a	32 (25%)
M1b	21 (16.5%)
M1c	35 (27.5%)

SSM, superficial spreading melanoma; NM, nodular melanoma; LMM, lentigo malignant melanoma; ALM, acral lentiginous melanoma; AJCC, American Joint Committee on Cancer.

### Methods

Blood samples (10 mL) were collected in EDTA tubes, and processed within 2 h of collection. ISET (Rarecells Diagnostics, Paris, France) was performed as previously described 24. Tumor cells (CTCs and/or CTMs) were identified and quantified in six May Grünwald Giemsa (MGG) stained membrane spots. Briefly, tumor cells were classified into three categories according to morphological criteria, as previously described: circulating nonhematological cells with malignant features (CNHC‐MF), CNHC with uncertain malignant features (CNHC‐UMF), and CNHC with benign features (CNHC‐BF) 24. As previously reported, morphological criteria for the identification of circulating melanoma cells with malignant features included: (1) cell size ≥16 *μ*m, (2) nucleo‐cytoplasmic ratio ≥50%, (3) irregular nuclear shape, (4) hyperchromatic nucleus, and (5) basophilic cytoplasm 12. A CTM was defined as a cluster of more than two circulating tumor cells. Immunocytochemistry (ICC) was performed on four membrane spots with anti‐PS100 (Roche Ventana, Tuczon), anti‐SOX10 (Cell Marque, Rocklin, CA), anti‐CD10 (Roche Ventana), and anti‐TRF2 (Novus Biologicals, Littleton, CO) antibodies. The protocols used for ICC on filters were previously described 13, 25. All spots were then reviewed independently by four cytopathologists (E.L., M.I., V.H., and C.B.) blinded to the diagnosis and clinical status of the patients. Immunohistochemistry (IHC) was performed on primary melanoma tumors using the four above‐mentioned antibodies.

The serum lactate dehydrogenase (LDH) biomarker level was measured 1 day before surgery and biopsy and on postoperative days 1 and 2 if patients were still hospitalized. Additionally, the LDH level was measured after the first line of treatment. The enzyme activity of LDH was analyzed routinely with a Roche Modular (Hitachi, Paris, France); normal levels of LDH were considered to be below the reference cutoff of 250 U/L.

### Statistical analysis

All calculations were performed with the statistical software R, a free software and environment for statistical analysis and graphics (version 2.9.0, Alcatel‐Lucent, Boulogne‐Billancourt, France). Spearman's correlation was used to correlate the CTC count with the serum LDH levels. Patient outcome and overall survival (OS) compared to the presence or absence of CTMs and/or iCTCs were assessed by Kaplan–Meier analysis with a log‐rank score for determining statistical significance. OS was defined as the interval between the diagnosis of metastatic melanoma and the date of death from any cause or the last follow‐up. A *P*‐value < 0.05 value was considered significant for all analyses.

## Results

**Figure 1:**
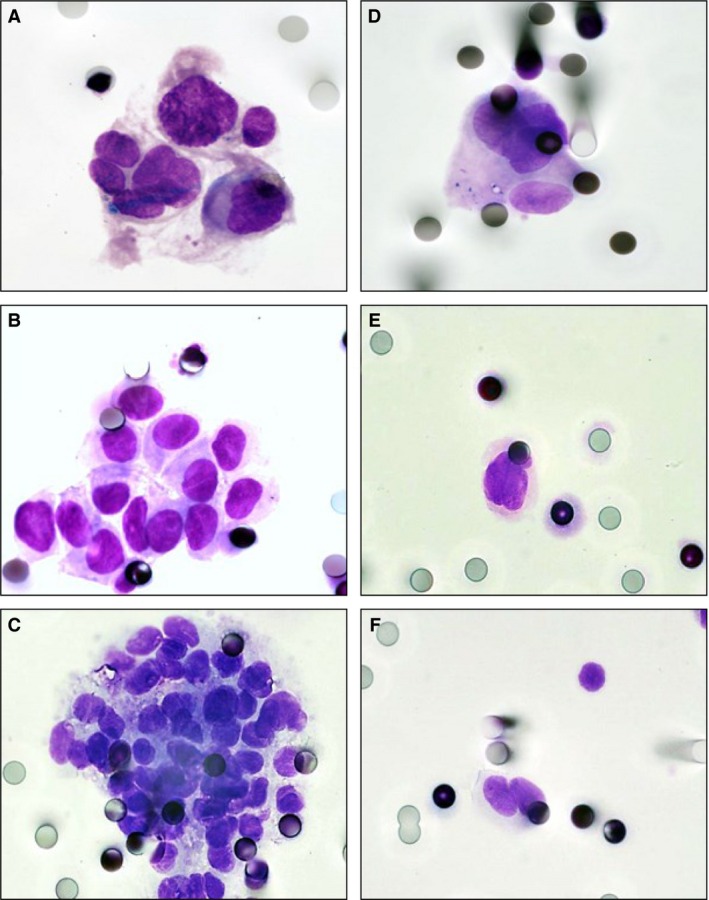
Morphological characteristics of circulating tumor microemboli (CTMs) and iCTCs in metastatic malignant patients. (A–C). CTMs of different size composed of CNHC‐MF (A) or of CNHC‐MF and CNHC‐MF (B and C). (D). iCTCs corresponding to CNHC‐MF. (E). isolated circulating tumor cells (iCTCs) corresponding to CNHC‐UMF. (F). iCTCs corresponding to CNHC‐BF detected in a patient with benign nevi. (A–F). MGG, original magnification, ×1000.

Of the 128 MMPs, 109/128 (85%) showed CTCs. 28/128 (22%) MMPs showed both CTMs and iCTCs, whereas 35/128 (27%) and 46/128 (36%) MMPs demonstrated CTMs and iCTCs alone, respectively. The number of CTMs varied from 3 to 9 per patient (mean: 4) and the number of iCTCs varied from 4 to 16 per patient (mean: 6). CTMs were composed of between three to fifty tumor cells (with a mean of 10 tumor cells per CTM) (Fig. 1A–C). Tumor cells forming the CTMs corresponded to CNHC‐MF (Fig. 1A) or to an association of CNHC‐MF and CNHC‐UMF (Fig. 1B and C). iCTCs were classified as CNHC‐MF in 59/65 (91%) patients and as CNHC‐UMF in 6/65 (9%) patients (Fig. 1D and E). Among the 37 patients with benign nevi, three patients showed CNHC‐BF (Fig. 1F).

**Figure 2:**
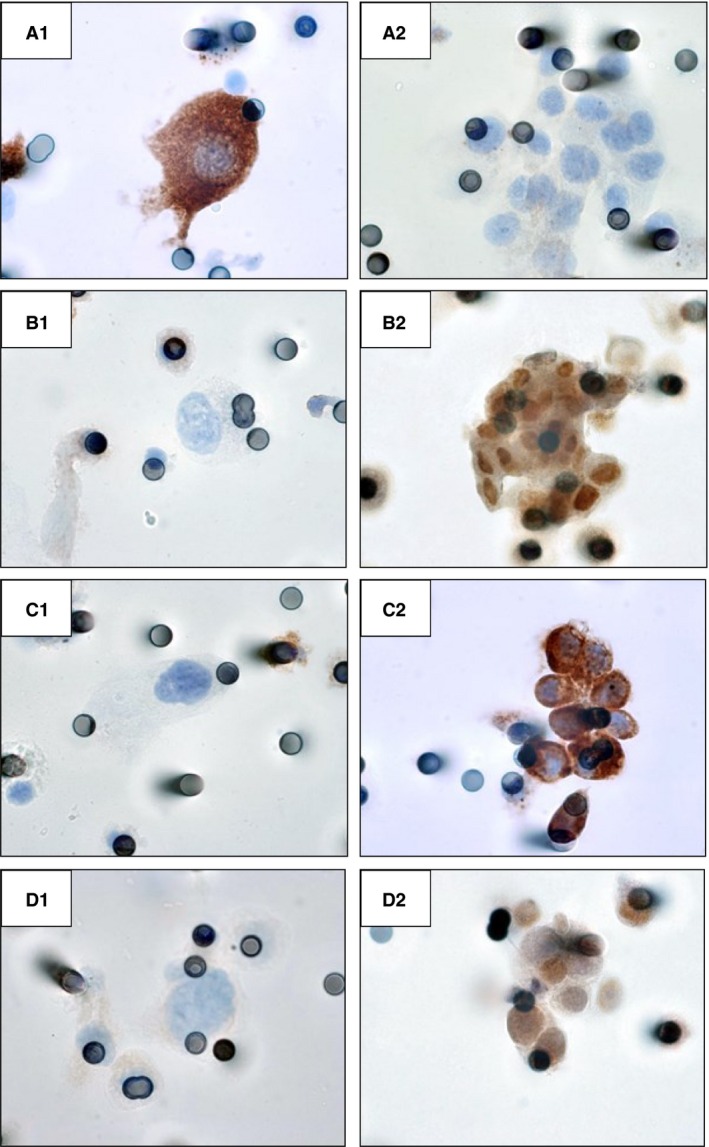
Different phenotypes of isolated circulating tumor cells (iCTCs) and circulating tumor microemboli (CTMs). A1–D1: immunostaining of iCTCs with anti‐PS100 (A1), anti‐TRF2 (B1), anti‐CD10 (C1), and anti‐SOX10 (D1) antibodies (immunoperoxidase, original magnification, ×100). A2–B2: immunostaining of CTMs with anti‐PS100 (A2), anti‐TRF2 (B2), anti‐CD10 (C2), and anti‐SOX10 (D2) antibodies (arrows: stained nuclei; arrowheads, stained cytoplasmic cell membranes; immunoperoxidase, original magnification, ×1000)

A homogeneous and strong cytoplasmic expression of PS100 was noted in all detected iCTCs (Fig. 2A1), whereas this expression was weaker, absent and/or heterogeneous in CTMs (Fig. 2A2). Conversely, TRF2, CD10, and SOX10 expression was weak or absent in iCTCs (Fig. 2B1, C1 and D1), whereas most of the tumor cells forming the CTMs strongly expressed these three markers (Fig. 2B2, C2 and D2). SOX10 and TRF2 and SOX10 were expressed in the nuclei, whereas CD10 was mainly expressed at the tumor cell membrane. The majority of corresponding primary melanoma tumors expressed strongly and diffusely PS100 and Melan A (Figure S1A and B). Conversely the intensity of SOX10, CD10 and TRF2 immunostaining was heterogeneous, depending on the tumor (Fig. S1C–H). CNHC‐BF stained strongly with the anti‐PS100 and the anti‐Melan A antibodies and was negative for the other antibodies. Finally none of the detected CTCs stained with the anti‐CD45 antibody, whereas the iCTCs and most of the CTMs stained with the anti‐Melan A antibody (Fig. S2).

**Figure 3:**
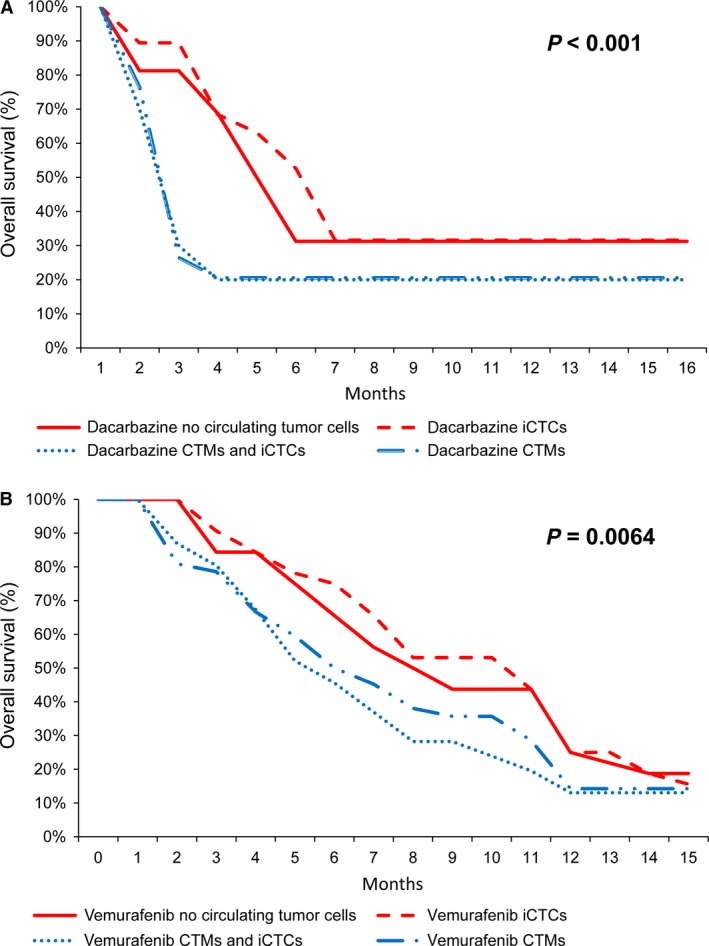
Kaplan‐Meier estimates of survival of MMPs according to the presence (blue curves) or the absence (red curves) of circulating tumor microemboli (CTMs) at baseline in patients treated with dacarbazine (A) or with vemurafenib (B).

Overall survival (OS) was significantly decreased in patients with CTMs alone or CTMs and iCTCs at baseline in comparison to patients with no CTMs or with iCTCs alone, independently of the therapeutic strategy (*P *<* *0.001 and *P *=* *0.0064, for dacarbazine‐treated patients and vemurafinib treatment patients, respectively) (Fig. 3A and B). OS was similar in patients without CTCs and with iCTCs alone at baseline (nonsignificant: *P *=* *0.37) (Fig. 3A and B). Elevated serum LDH levels were found in 27.5% and 5.4% of patients when measured before and after the first line of treatment. Interestingly both iCTCs and CTMs count strongly correlated with serum LDH levels (*P *<* *0.001, Fig. S3). The LDH levels were only associated with OS in a univariate analysis when measured before the treatment (HR 2.49, 95% CI 1.27–4.87, *P *=* *0.01). A multivariate Cox regression model showed that the presence of CTMs at baseline (*P *=* *0.022), along with elevated serum LDH levels before treatment (*P *=* *0.02) and extension of disease (stage M1a vs. stage III, stage M1b vs. stage III, and stage M1c vs. stage III) were independent predictors of poor OS (Table 2).

**Table 2:** Cox multivariate regression analysis of prognostic factors for overall survival in 128 MMPs

	Multivariate		
Variable	HR^a^	95% CI^b^	*P‐*value^c^
Breslow	14.3	0.3–877.5	0.249
Ulceration	4.4	1.81–9.4	0.001
Mitotic rate	2.2	0.7–6.8	0.290
Elevated LDH	2.81	1.25–5.62	0.02
M1a	4.1	1.81–6.4	0.01
M1b	4.4	1.7–6.6	0.01
M1c	5.2	1–21	0.002
CTMs (circulating tumor microemboli)	5.1	2–19	0.022

a HR: hazard ratio.

b CI: confidence interval.

c *P*‐value < 0.05 statistically significant.

## Discussion

This work showed that tumor cells are frequently detected in blood of MMPs by ISET as both iCTCs and CTMs. The presence of CTMs was shown to be a negative prognostic factor in these patients. Interestingly, and more particularly, among the patients treated with anti‐*BRAFV600E* therapy, the presence of CTMs (with or without iCTCs) at baseline and after the first line of treatment was an independent negative prognosis factor. Previous studies have demonstrated that ISET can be a good approach for detection and quantification of CTCs in MMPs 12, 14. Moreover, a recent study using this technology has shown that CTCs detected in MMPs can be separated into two different subpopulations, iCTCs and CTMs 14. It has been shown that the number of CTCs detected in MMPs correlated with OS 14. However, the question concerning the importance and the impact of the respective CTM and the iCTC populations as a prognostic factor were not addressed. In our study the presence of iCTCs alone was not a prognostic factor since OS was almost similar in treated patients with or without iCTCs at baseline.

Interestingly, the number of CTMs decreased in some patients after the first line of treatment using targeted therapy against a *BRAFV600E* mutation. In some patients, the CTMs were no longer detected after the first line of treatment. However, in a couple of patients CTMs were still present after treatment, even if globally their number was decreased. The OS of MMPs treated with the targeted therapy against a *BRAF* mutation was better when no CTMs were noted after the treatment, and independently of the number of CTMs detected at baseline (not shown). Since the assessment of the *BRAFV600E* status can be detected by ICC on CTCs, further study is in progress to look for the expression of BRAF in CTMs and to compare this expression in iCTCs 13.

By using four different antibodies, we demonstrated that different subpopulations of melanoma cells were present among the CTMs, with a different phenotype to that of iCTCs. PS100, a sensitive and quite specific marker of differentiated melanoma cells, was strongly expressed in most iCTCs 18. Conversely, only few CTMs showed staining with the anti‐PS100 antibody. These results may point to the presence of more poorly differentiated melanoma cells among the CTMs. When looking for the expression of SOX10 and CD10 in iCTC subpopulations, the staining was very variable depending on the subtype of these populations. SOX10 and CD10 were strongly expressed in a large majority of CTMs, whereas almost all iCTCs were negative or expressed at a low level these molecules. In this context it was recently highlighted that SOX10 expression in melanoma was associated with more tumor aggressiveness and invasiveness 20, 21, 26. Similarly, CD10 expression in melanoma was demonstrated to be representative of a subpopulation of aggressive cells associated with poor patient outcome 22. The present results underlined that the tumor cells forming CTMs could have a more aggressive phenotype and a high metastatic potential. The expression of TRF2 was also variable depending on the population of CTCs. TRF2 was strongly expressed in CTMs and weakly or not expressed in iCTCs. Interestingly, TRF2 is considered to be expressed in aggressive and invasive tumors, including melanoma, so the present results may identify a subset of aggressive TRF2‐positive melanoma cells among the CTMs 23, 27. Interestingly, TRF2 is also able to regulate the activity of natural killer cells independently of its role in telomere protection 28. It will thus be interesting in a future study to see whether such a role for TRF2 is part of a general mechanism by which CTCs bearing telomere damage are eliminated by the innate immune system. Finally, molecules targeting TRF2 could represent valuable multimodal drugs that combine different therapeutic activities in one single component, with obvious advantages in terms of simplicity of treatment and selectivity for melanoma cells. By looking at the expression in CTCs of other molecules than PS100, SOX10, CD10, and TRF2 (e.g., MITF, Melan‐A, high molecular melanoma‐associated antigen, CD271 and MAGEC) it was also observed that the phenotype of CTCs was very variable depending on the subtype of CTCs (iCTCs vs. CTMs) 14. Taken together, the previously published and present data show that the melanoma cells of CTMs had a particular phenotype, which could allow them to be more metastatic.

In addition, more significant power to discriminate between low and high risk of a specific outcome could be obtained by combining multiple biomarkers, such as LDH levels with baseline CTCs, as shown in other types of cancer. Our data suggest that increased LDH levels, as an indicator of tumor burden, may precede CTC shedding into the blood flow 29.

Interestingly, when detected with ISET, the CTMs were often associated with blood cells, in particular with aggregates of platelets and neutrophils (not shown) and this phenomenon could allow the CTCs to better survive the blood pressure and/or anoikis. In this regard, previous studies have shown that the presence of platelets, neutrophils, and CTCs can improve the survival of CTCs in the blood stream of patients and may create a favorable microenvironment for onset of metastasis 30, 31.

Taken together this study reports that the presence of CTMs in blood from MMPs is synonymous of a more aggressive behavior and a higher metastatic potential compared to MMPs where only iCTCs are isolated in the blood stream. Indeed, the detection of iCTCs alone at baseline is not associated with worse OS. This could reflect the possibility that iCTCs are less metastatic because they could not survive in the blood stream. In this regard, higher expression of TRF2, CD10, and SOX10 in CTMs was found to correlate with worse prognosis in MMPs, whereas these molecules were only weakly or not expressed in iCTCs. Finally depending on the level of certain molecules, in particular TRF2, CTCs can be more or less aggressive and susceptible to apoptosis.

## Conflict of interest

The authors state no conflict of interest.

## Supporting information


**Figure S1.** Different phenotypes of the primary cutaneous melanoma. A–H: Immunostaining with anti‐PS100 (A), anti‐Melan A (B), anti‐SOX10 (C and D), anti‐CD10 (E and F), and anti‐TRF2 (G and H) antibodies (immunoperoxidase, original magnification, ×200).

**Figure S2.** iCTCs and CTMs immunostaining with anti‐Melan A and anti‐CD45 antibodies

**Figure S3.** Positive correlation between (A) serum LDH levels and iCTC count, and (B) serum LDH levels and number of CTMs in metastatic malignant patients.
