# Supplementary figures and images for: High expression of TRF2, SOX10, and CD10 in circulating tumor microemboli detected in metastatic melanoma patients. A potential impact for the assessment of disease aggressiveness
Source: Cancer Med. 2016 Mar 6;5(6):1022–30. doi: 10.1002/cam4.661 (PMC4924359; doi:10.1002/cam4.661)

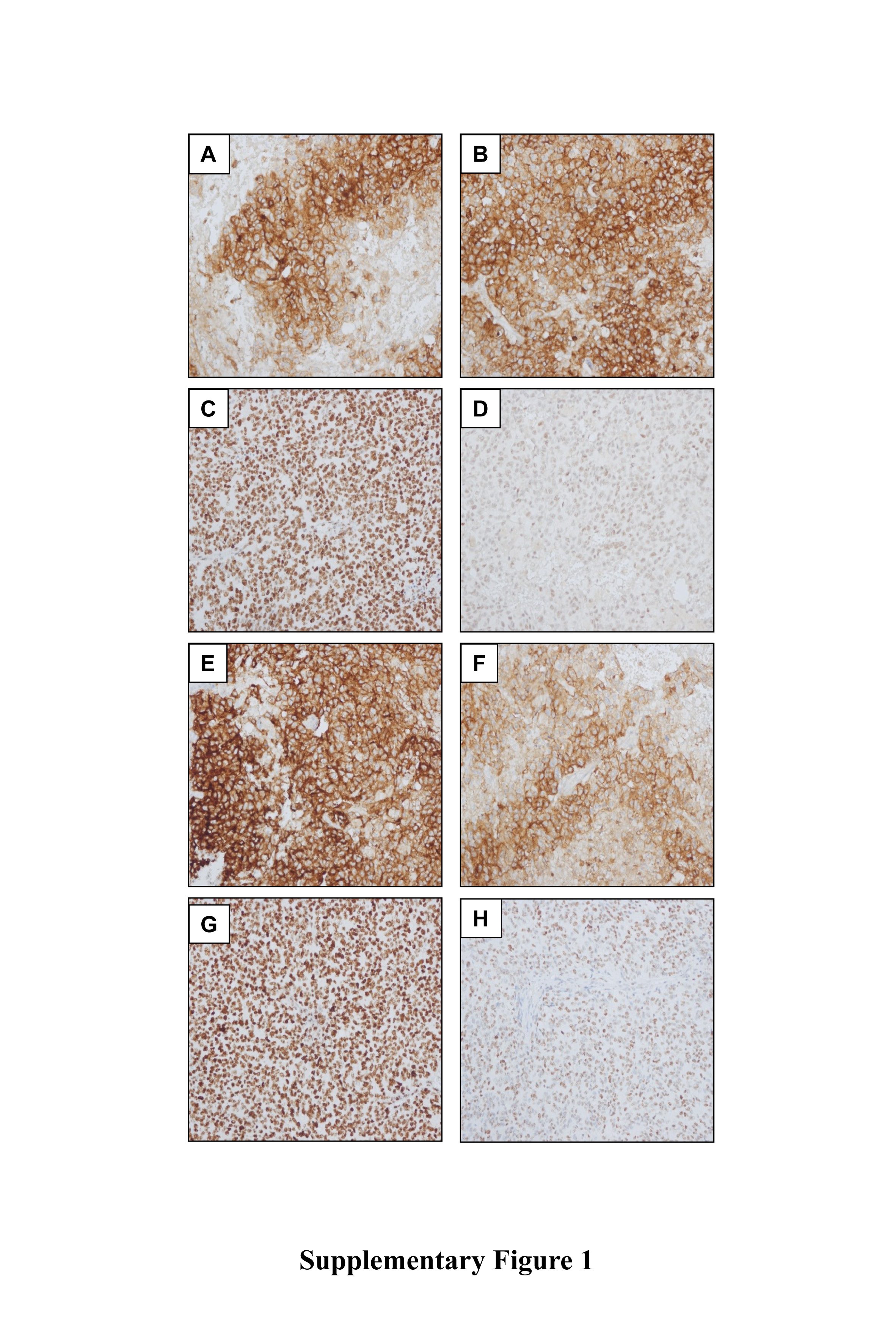

Supplement: Supplementary file 1 — Figure S1. Different phenotypes of the primary cutaneous melanoma. A–H: Immunostaining with anti‐PS100 (A), anti‐Melan A (B), anti‐SOX10 (C and D), anti‐CD10 (E and F), and anti‐TRF2 (G and H) antibodies (immunoperoxidase, original magnification, ×200). [file CAM4-5-1022-s001.tif]

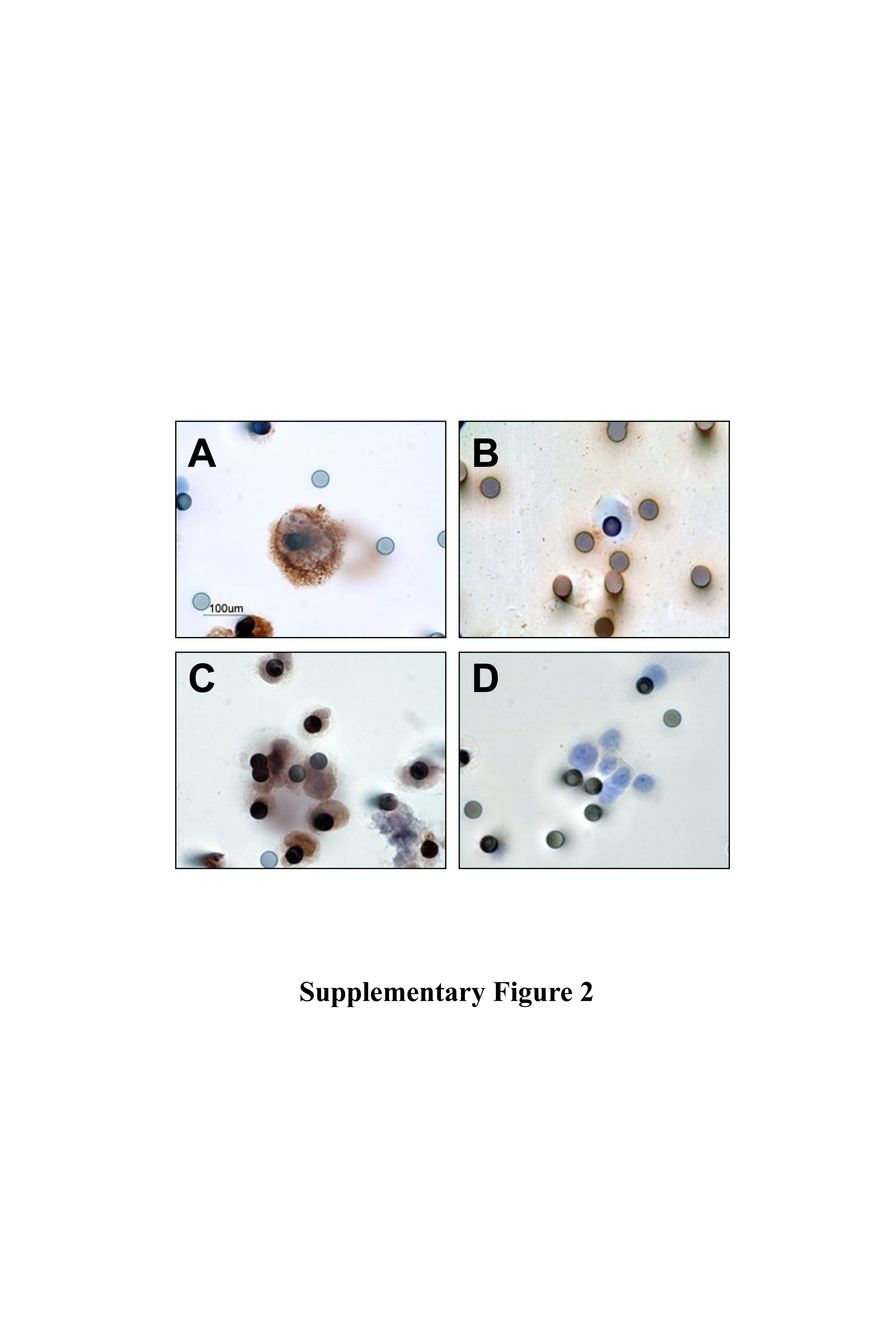

Supplement: Supplementary file 2 — Figure S2. iCTCs and CTMs immunostaining with anti‐Melan A and anti‐CD45 antibodies [file CAM4-5-1022-s002.tif]

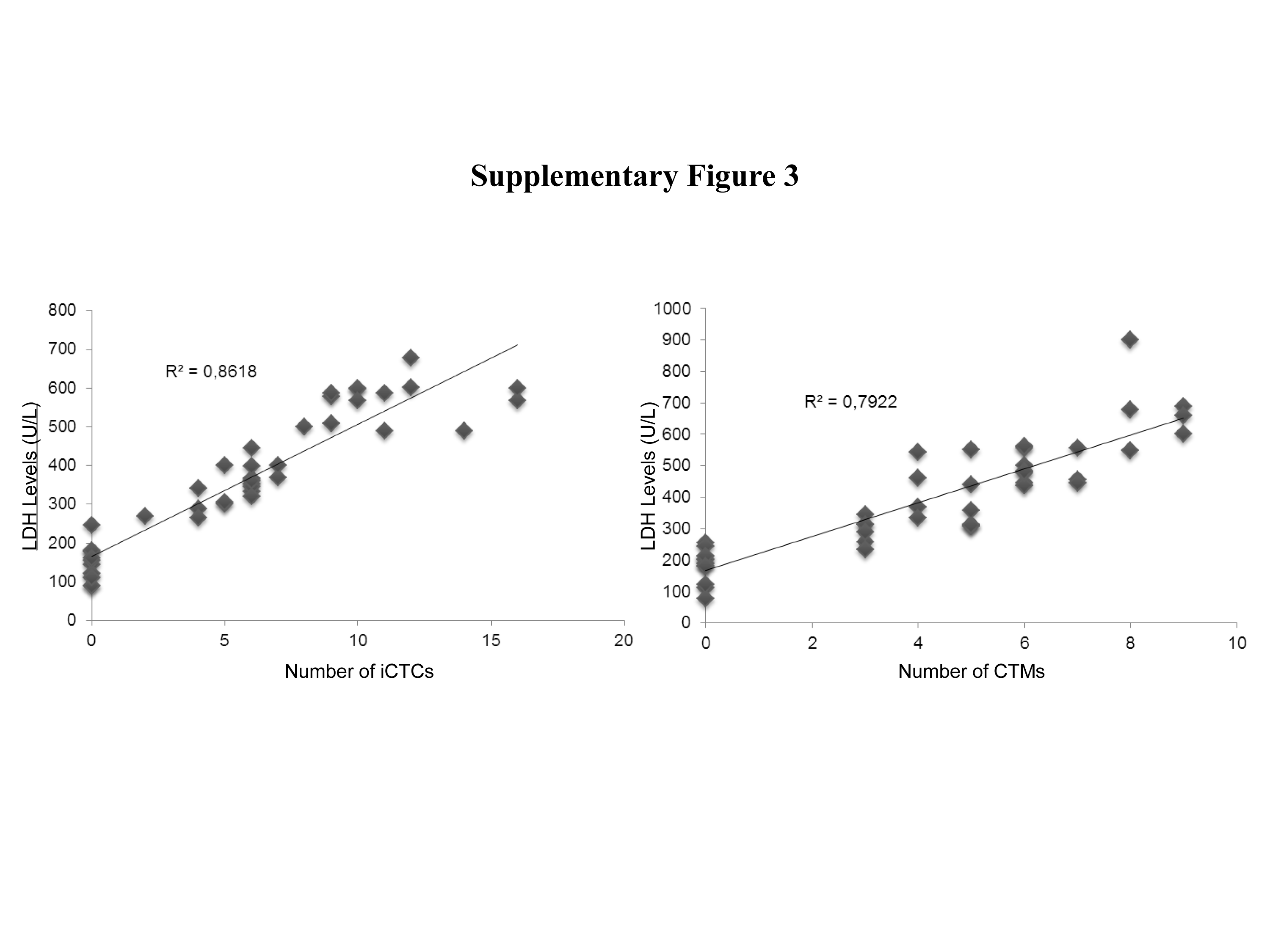

Supplement: Supplementary file 3 — Figure S3. Positive correlation between (A) serum LDH levels and iCTC count, and (B) serum LDH levels and number of CTMs in metastatic malignant patients. [file CAM4-5-1022-s003.tif]
